# Nano-Architecture of nitrogen-doped graphene films synthesized from a solid CN source

**DOI:** 10.1038/s41598-018-21639-9

**Published:** 2018-02-19

**Authors:** Chiranjeevi Maddi, Florent Bourquard, Vincent Barnier, José Avila, Maria-Carmen Asensio, Teddy Tite, Christophe Donnet, Florence Garrelie

**Affiliations:** 1 Univ Lyon, Université Jean Monnet, Laboratoire Hubert Curien UMR 5516, F- 42000 Saint-Étienne, France; 2 Laboratoire Georges Friedel, Ecole Nationale Supérieure des Mines, F-42023 Saint-Etienne, France; 3 Synchrotron SOLEIL & Université Paris-Saclay, Saint Aubin, F-91192 Gif sur Yvette France

## Abstract

New synthesis routes to tailor graphene properties by controlling the concentration and chemical configuration of dopants show great promise. Herein we report the direct reproducible synthesis of 2-3% nitrogen-doped ‘few-layer’ graphene from a solid state nitrogen carbide a-C:N source synthesized by femtosecond pulsed laser ablation. Analytical investigations, including synchrotron facilities, made it possible to identify the configuration and chemistry of the nitrogen-doped graphene films. Auger mapping successfully quantified the 2D distribution of the number of graphene layers over the surface, and hence offers a new original way to probe the architecture of graphene sheets. The films mainly consist in a Bernal ABA stacking three-layer architecture, with a layer number distribution ranging from 2 to 6. Nitrogen doping affects the charge carrier distribution but has no significant effects on the number of lattice defects or disorders, compared to undoped graphene synthetized in similar conditions. Pyridinic, quaternary and pyrrolic nitrogen are the dominant chemical configurations, pyridinic N being preponderant at the scale of the film architecture. This work opens highly promising perspectives for the development of self-organized nitrogen-doped graphene materials, as synthetized from solid carbon nitride, with various functionalities, and for the characterization of 2D materials using a significant new methodology.

## Introduction

Graphene, a two-dimensional carbon material, consists of a single layer of sp^2^-hybridized carbon atoms arranged in a honeycomb lattice^1,2^. In recent years, graphene has attracted an enormous amount of attention thanks to its extraordinary electronic, optical, mechanical and thermal properties, which make it a promising material for a wide range of applications, including energy conversion and storage, electrochemistry, electronics, sensors and solar cells^3–8^. Recently, Ferrari *et al*.^9^ published a roadmap for graphene, highlighting both fundamental and technological challenges in the field. The authors focus on different ways to synthesize tailored graphene properties. Among the properties that need to be fully controlled in many applications are electronic properties, and synthesizing remains challenging.

Theoretical and experimental studies have revealed that the semi-conductor properties of graphene-based materials can be controlled by doping graphene materials with heteroatoms, including nitrogen^10–13^. Introducing dopants in graphene can tune the electronic properties of the modified material and change it from an n-type into p-type semiconductor. Such a perspective is encouraged by the story of nitrogen doped carbon nanotubes (N-CNT), which greatly extended the applications of such a material^14–16^.

In recent years, several approaches have been proposed to synthesize nitrogen doped graphene (NG) films either by direct synthesis, including chemical vapor deposition^17–20^, segregation growth^21^, solvothermal process^22^, arc discharge^23^, or by post-synthesis treatment, including thermal treatments^24,25^, plasma treatments^10,26–28^, or hydrazine hydrate treatments^29^. The direct synthesis methods have the potential to create higher homogenous doping within the bulk graphene than post-synthesis treatments, which only lead to surface doping. Most of these methods enable the incorporation of nitrogen contents within the first at.%, with various bonding configurations including pyridinic N (two C-N bonds in a hexagon), pyrrolic N (two C-N bonds in a pentagon) and quaternary N (three sp^2^ C-N bonds) ones. The advantages of nitrogen-doped graphene have been highlighted in many recent papers, including better electrochemical activities than noble metal catalysts to reduce CO_2_ gas^30^, almost double reversible discharge capacity compared to pristine graphene due to the large number of surface defects induced due to doping by nitrogen^31^, a high activity rate, long-lasting stability and outstanding crossover resistance for electrocatalysis of oxygen reduction reaction in alkaline medium^32^. According to a review by Wang *et al*.^12^, the main challenges remain the need for large-scale N-doped graphene materials, as well as better control of the nitrogen concentration, bonding and depth distribution within the graphene material. Our paper focuses on such objectives by exploring the ability of pulsed laser deposition (PLD) to synthetize solid nitrogen-carbon sources as precursors for nitrogen-doped graphene films. PLD has been less well studied than other methods to obtain graphene films, but is a versatile deposition method, known to enable a wide range of film nanostructures and compositions, sometimes beyond the limit of solubility^33^. This approach may be particularly fruitful for the synthesis of solid carbon graphene precursors containing a wide range of heteroatom concentrations, and thereby making it possible to study their catalytic transformation into doped graphene films.

## Results and Discussion

### Synthesis route

To prepare NG films, we exploited our recent success^34,35^ by depositing few-layer self-organized pure graphene (fl-graphene) by vacuum thermal annealing, in the presence of a metallic Ni catalyst, of a solid-state carbon source constituted of an amorphous carbon film (a-C). We previously showed that a femtosecond laser is particularly suited to deposit very thin layers of a-C, with extremely low internal stress^36^ and an architecture of predominantly Csp^2^ rings^37^. The carbonaceous films can be converted into graphene by *in situ* vacuum thermal annealing in the presence of a nickel thin film catalyst. Moreover, PLD, optionally combined with plasma assistance, has also been shown to be capable of incorporating up to 28 at.% of nitrogen in the a-C film to form an amorphous nitrogen-containing a-C:N film, by ablating the carbon source in the presence of nitrogen gas^38–40^. The fl-graphene films have previously been shown to be highly efficient as active substrates for molecular diagnosis by surface-enhanced Raman spectroscopy (SERS) and for electrografting, to develop new high-performance electrodes for electrochemical detection^41^.

Our NG films were obtained in a three-step process, (i) synthesis of a-C:N films by femtosecond pulsed laser ablation, (ii) synthesis of a super-imposed metallic nickel film by thermal evaporation, and (iii) thermal annealing in vacuum, to generate the nitrogen-doped graphene films on the top surface of the Ni catalyst. Our nanoscale investigations of the architecture and chemistry of NG combined classical analyses with an original Auger mapping investigation, to the best of our knowledge never previously undertaken, which provided significant insights into the development and control of graphene-based thin layers.

**Figure 1:**
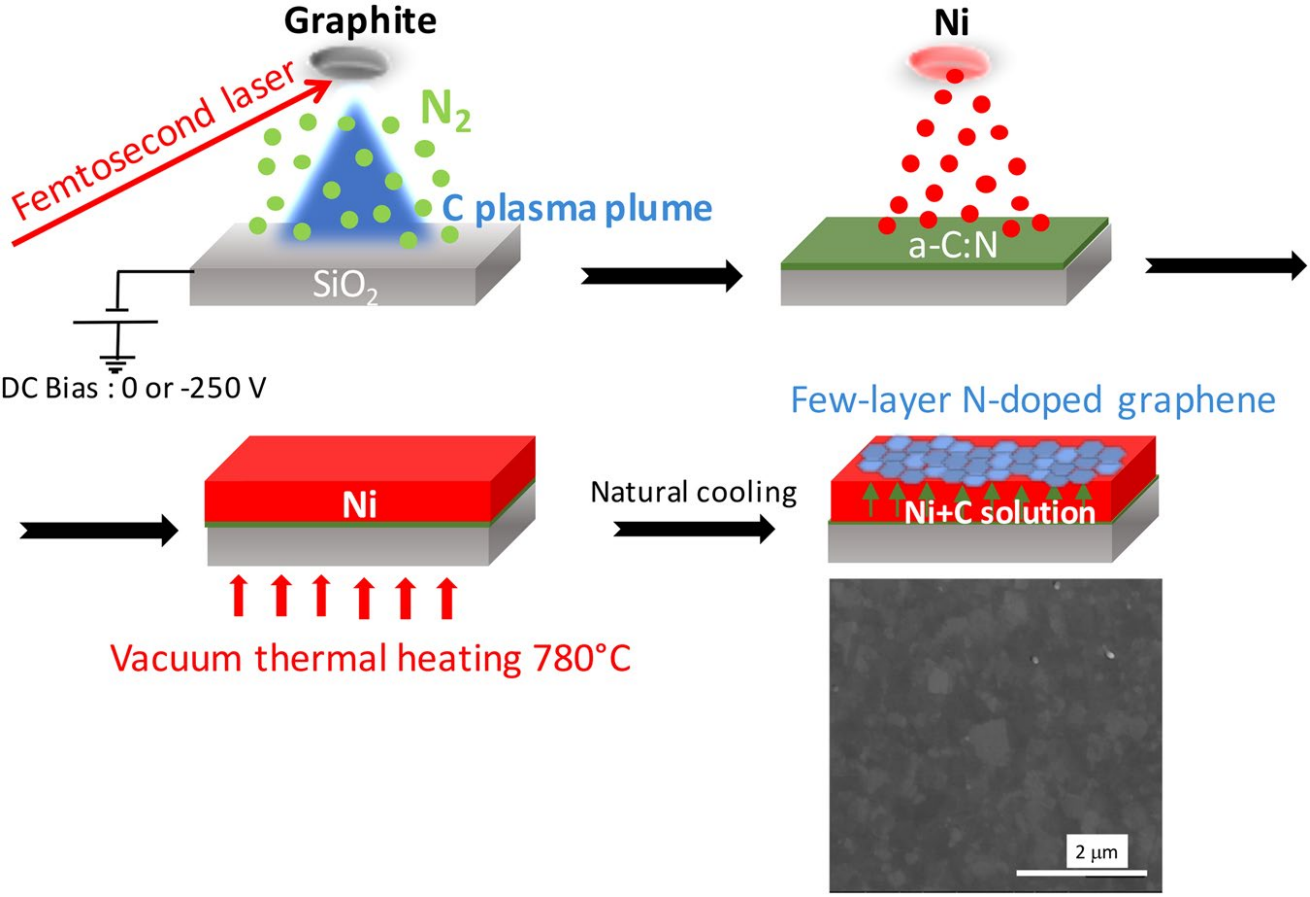
Synthesis route of few-layer N-doped graphene films obtained by femtosecond pulsed laser deposition. A femtosecond laser beam is focused on a graphite target in vacuum, to form a carbon-based plasma expanding in an atmosphere of gaseous nitrogen. The deposited a-C:N film (10 nm thick, N content depends on the N_2_ pressure and substrata bias) is next covered by a nickel film (150 nm) by vacuum thermal evaporation. The a-C:N/Ni sandwich is then heated to 780 °C in vacuum for 30 minutes with a heating ramp of 4 °C.mn^−1^ followed by natural vacuum cooling for 5 hours, thereby forming few-layer N-doped graphene on the top surface. The SEM picture is related to the NG2 sample (2.0%N). Note that all N-doped graphene surfaces have a similar mottled appearance.

NG films were synthesized by vacuum annealing of a sandwich type of Ni/a-C:N architecture deposited on a SiO_2_ substrate. The amorphous carbon nitride (a-C:N) and Ni catalyst layers were synthesized successively by femtosecond pulsed laser deposition (fs-PLD) and thermal evaporation, respectively. A reference a-C film with no nitrogen incorporation was also synthesized. Fig. 1 gives a step-by-step view of the synthesis route.

A 10 nm thick amorphous carbon nitride (a-C:N) thin film was prepared by focusing the femtosecond laser beam on a high purity graphite target, with a constant energy density. Nitrogen was incorporated in the carbonaceous films by introducing nitrogen gas at pressures of 0.5, 1 and 10 Pa during laser irradiation. An additional nitrogen pressure of 5 Pa was coupled with a substrate bias of −250 V to increase the nitrogen content in the film through the reactivity of the plasma. An increase in nitrogen pressure from 0.5 to 10 Pa without bias assistance increases the nitrogen content in the a-C:N films from 4 to 16 at.%. Additional bias associated with the nitrogen gas pressure of 5 Pa makes it possible to reach a nitrogen content of 21 at.%. An increase in nitrogen gas pressure of more than 5 Pa with bias assistance will not increase the nitrogen content in the film higher than the previous value. The present set of experiments thus allowed us to explore a-C:N films as N-doped graphene precursors, with an initial nitrogen content ranging from the 4–21 at.%. Following deposition of the carbon film, a 150 nm thick nickel film was deposited by thermal evaporation on top of the a-C(:N) film. The thicknesses of 10 nm for a-C:N and 150 nm for Ni were fixed based on previous published works, including ours, on the synthesis of graphene on a “substrate/a-C/Ni” of similar architecture, followed by thermal heating^35,42–46^. The thickness of a-C film usually ranges between 2 and 40 nm (and even 80 nm in the case of a-C:Si:H film), whereas the thickness of Ni superimposed films range between 20 and 550 nm, but most between 100 and 200 nm. Consequently, we chose 10 nm for the a-C:N and 150 nm for the Ni film, to be as close as possible to the most widely published conditions with similar film architecture but without nitrogen. According to Koh *et al*.^47^, for the formation of graphene from PLD, a Ni catalyst is preferable, due to the adequate combination of carbon solubility and diffusion rate, compared to the other metals Cu, Co and Fe. The a-C(:N)/Ni films were further annealed in vacuum at 780 °C for 30 minutes and cooled down naturally to room temperature. Whatever the nature of the original a-C and a-C:N films, Fig. 1. shows the mottled appearance along with a low surface roughness of 5–10 nm as evidenced by SEM. This appearance may be consistent with the formation of graphene sheets on the top surface of the grains of the nickel catalyst during thermal annealing followed by the cooling process. Indeed, based on previous studies^48^, our process is consistent with a three-step growth synthesis involving (i) dissociation of solid carbon thin film, (ii) dissolution and diffusion of carbon/nitrogen in the metal during heating at high temperature, and (iii) segregation and/or precipitation of carbon/nitrogen atoms at the surface of the metal either at the growth temperature when the solid solution has been saturated, or during cooling because of the decrease in carbon solubility of the metal.

### Chemistry of the N-doped graphene films

The investigation of the chemical forms of carbon and nitrogen in N-doped graphene is critical for the control of the film properties. The nitrogen concentration in the N-doped graphene films, as deduced from XPS, ranged from 2.0 to 2.9 at.%, see summary in Table 1. Thus, in our experimental range, the concentration of nitrogen in the a-C:N precursor does not notably influence the final nitrogen concentration in the N-doped graphene films.

**Table 1:** Nitrogen concentration, deduced from XPS, in the a-C:N films deposited by femtosecond laser ablation before annealing, and in the graphene films after thermal annealing.

Samples	N/(N + C) % in a-C:N films before thermal annealing	N/(N + C) % in graphene films after thermal annealing
Graphene	0	0
NG1	4	2.0
NG2	10	2.2
NG3	16	2.4
NG4	21	2.9

Indeed nitrogen dissolution and/or diffusion in the nickel layer could be much lower than for carbon. Thus, the variability of nitrogen concentration in the a-C:N films cannot be considered as a key to obtaining a wide range of nitrogen contents in N-doped graphene, using the experimental parameters investigated here.

**Figure 2:**
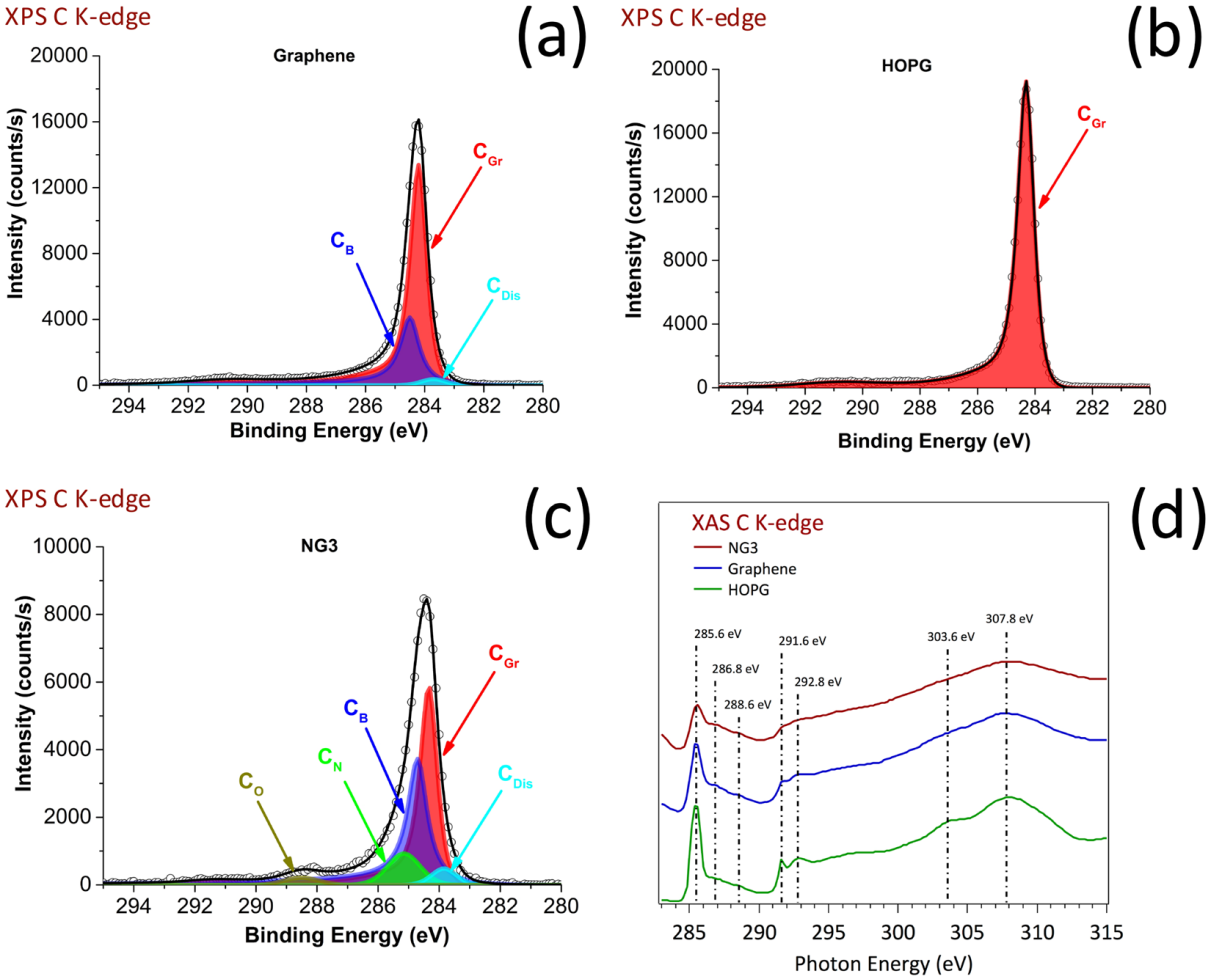
Investigation of the carbon chemistry of the graphene layers. (**a**) XPS C K-edge of the undoped graphene; (**b**) XPS C K-edge of the HOPG reference; (**c**) XPS C K-edge of the NG3 N-doped graphene (2.4%N); (**d**) XAS C K-edge of the HOPG, undoped graphene and NG3 N-doped graphene.

Figure 2a shows the C1 photoelectron signal of the pure graphene film, used as undoped reference. Considering the unique C1 contribution measured on the HOPG reference (C_Gr_) and depicted in Fig. 2b, the carbon in pure graphene mainly comprises C_Gr_ (284.3 eV ± 0.1 eV, 69%). Two other contributions, labelled C_B_ (284.7 ± 0.1 eV, 29%) and C_Dis_ (283.7 ± 0.1 eV, 2%) were also detected.

**Figure 3:**
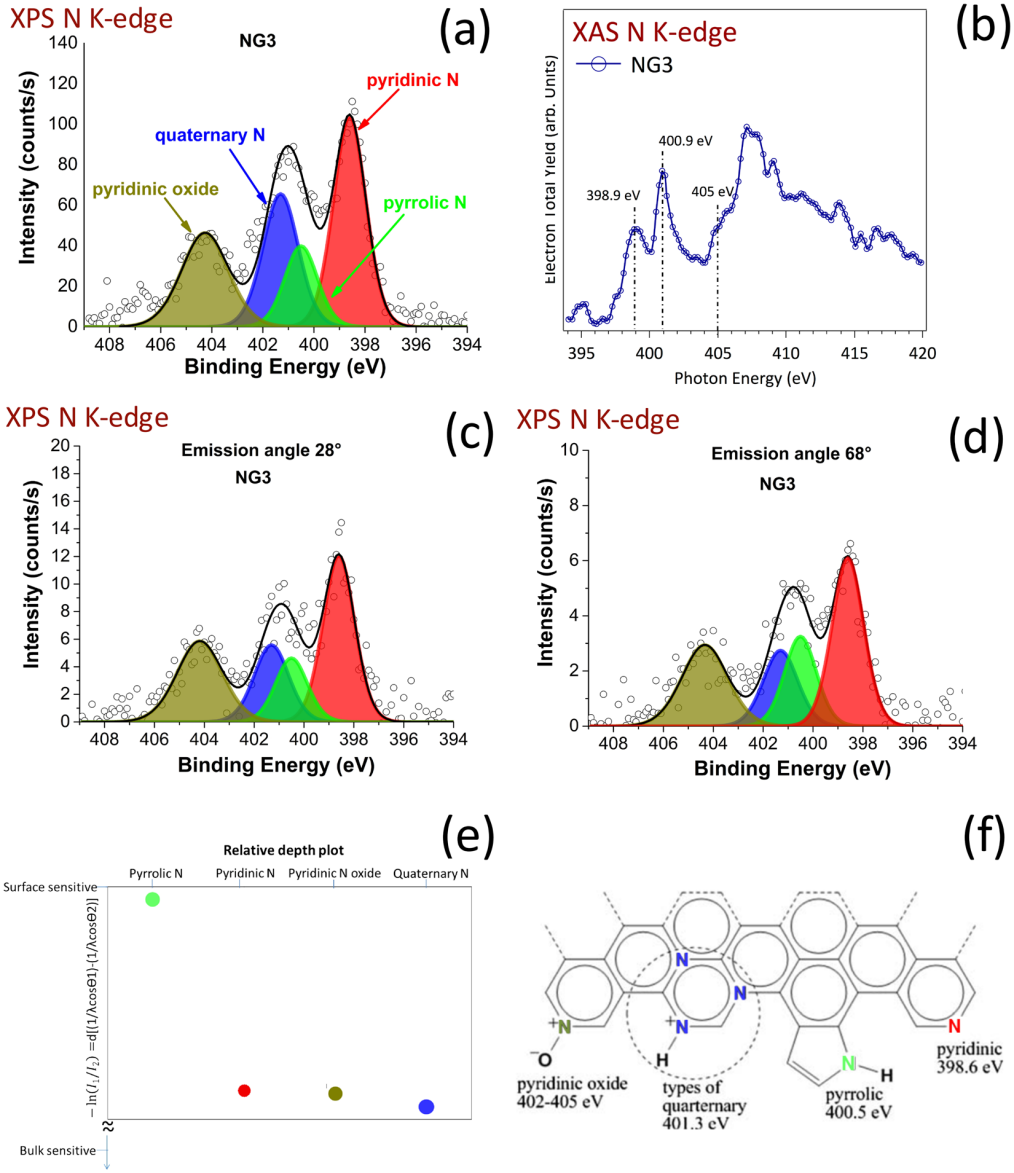
Chemical investigation of the nitrogen chemistry of the graphene layers. (**a**) XPS N K-edge of the NG3 N-doped graphene (2.4%N); (**b**) XAS N K-edge of the NG3 N-doped graphene; (**c**) ARXPS N K-edge of the NG3 N-doped graphene, with an emission angle of 28°; (**d**) ARXPS N K-edge of the NG3 N-doped graphene, with an emission angle of 68°; (**e**) Ratio of intensity I_1_ (from ARXPS with 68°) to intensity I_2_ (from ARXPS with 28°) for each of the four nitrogen chemical contributions deduced from XPS, giving rise to a surface predominance of the pyrrolic from (green signal); (**f**) molecular scheme of the various N chemical forms identified by XPS and XAS in a N-doped graphene monolayer.

Their assignments are based on previous interpretations of graphene films obtained using metallic catalysts^49^. C_Gr_ is related to graphitic (Csp^2^ hybridized) carbon. C_B_ is related to deleterious (Csp^3^ hybridized) carbon on carbon at the periphery of the graphene domains. The origin of the C_Dis_ corresponds to residual carbon dissolved in solid solution in the Ni catalyst. Such a contribution can be attributed to the “transparency” of the graphene layers (4 layers represent a thickness of less than 2 nm) to C1 photoelectrons emitted by carbon atoms dissolved in the nickel catalyst close to the Ni/graphene interface, and/or to residual areas of the surface not fully covered by the graphene film. The C1 contributions in the four N-doped graphene films were similar, and only the case of the NG3 films is depicted in Fig. 2c. The three chemical forms of carbon observed with the pure graphene were still present, but the C_Gr_/C_B_ ratio decreased from 2.38 for the pure graphene to an average of 1.09 (standard deviation 0.1), due to the presence of N atoms scattered over the graphene sheets. Ten percent of the carbon atoms were bonded to nitrogen (C_N_, 285.2 ± 0.5 eV) and a residual 2–3% was bonded to oxygen (C_O_, 288.4 ± 0.5 eV) which was not detected in the pure graphene. Wang *et al*.^26^ reported similar results, indicating that oxygen may be chemisorbed in the presence of pyrrolic and pyridinic nitrogen, which could be an advantage for some applications, such as fuel cells. Concerning the XAS investigations, Fig. 2d shows the spectra of the carbon K-edge of HOPG, graphene and NG3 films. The distinctive spectroscopic characteristics provide interesting insight into the systems investigated and their attribution fully supports our XPS results. In particular, the peaks at 285.6 eV and 291.6 eV correspond to the excitation of the C1 core electrons, respectively in the π* and σ* states in the conduction band^50^. They These peaks were attributed to the sp^2^ hybridization of graphitic carbon, in agreement with the graphitic component (C_Gr_) quantified by XPS. These peaks dominate in HOPG. The XAS spectra of both graphene and NG3 also showed a significant increase in two other small features located in the C1s→π* region (286.8 and 288.6 eV), corresponding to interlayer states, whose interpretation is still under debate in the literature. They are frequently attributed to functionalization (C-O, C-H bonding surface contamination) or to the dispersion of three dimensional unoccupied bands in graphite^51^. It is interesting to note that the contribution of these interlayer states is higher in graphene than in HOPG, and in NG3 than in graphene, at the expense of a decrease in the C1s→π* and C1s→σ* peaks. Thus, the interlayer states may can be attributed to the C_B_ carbon localized at the periphery of graphene domains, as detected by XPS (Fig. 2a). The contribution of the interlayer states continued to increase in the NG3 sample whose XPS analysis revealed a higher C_B_ contribution as well as additional C_N_ and C_O_ contributions (Fig. 2c**)**. Thus, the interlayer states mainly detected at 286.6 and 288.6 eV can both be attributed to edge carbon or carbon bonded to nitrogen or oxygen, without being able to distinguish their respective contributions more precisely at the present stage of investigation. By comparing the structure of carbon spheres with and without nitrogen concentrations in the same range as our graphene films (2.5–3.5%), using XAS, Ray *et al*.^52^ recently observed interlayer states at 287.6 and 288.6 eV they attributed to C-H, C=O and C=N bonds, in agreement with our observations. The XAS and XPS N1s spectra revealed even more precise information on the type of bonding between C and N. As the N1s spectra for the four different nitrogen concentrations of N-doped graphene films were similar, only the case of the NG3 films is depicted in Fig. 3.

XPS and XAS spectra are depicted in Fig. 3a and Fig. 3b, respectively. Based on previous attributions of N-doped graphene by XPS analysis^12,21,26,28,53^ and by XAS analysis^54–57^, a predominant pyridinic contribution (35–41% deduced from XPS) was detected at 398.6 ± 0.5 eV by XPS, at 398.9 ± 0.5 eV by XAS. The relative preponderance of pyridinic nitrogen is particularly interesting for highly active and stable electro-catalysts dedicated to oxygen reduction activity^32^. A contribution centered at 401.0 ± 0.5 eV (XPS) and 400.9 ± 0.5 eV (XAS) may correspond to N bonded to C in amine/amide groups, or in various types of quaternary N incorporated in six-fold aromatic cycles, including pyrimidine. The XPS peak may be split into two contributions, as observed by Wang *et al*.^12^, with a lower contribution at 400.7 ± 0.5 eV corresponding to pyrrolic N, and a higher contribution at 401.4 eV ± 0.5 eV corresponding to quaternary N. Another contribution centered at 404.3 eV ± 0.5 eV (XPS) and 405.0 ± 0.5 eV (XAS) may correspond to either amino groups including saturated heterocyclic amine (up to 3 N incorporated in the six fold ring), or to pyridinic oxide. Since our concentration of nitrogen in N-doped graphene was rather low and considering the detection of oxygen by XPS, the presence of pyridinic oxide was suspected. The XAS contribution at binding energies higher than 407 eV corresponds to the continuum of the XAS spectrum of nitrogen. The complete XPS N1s results and interpretation related to all films are reported in Table S3, with no significant differences observed between the different N-doped graphene films. ARXPS investigations revealed a slight relative increase in the pyrrolic form on the top surface of the N-doped graphene films. Figure 3c and d exhibit the N1s spectra of NG1 with an emission angle of 28° (more “bulk” sensitive) and 68° (more “surface” sensitive) respectively, making it possible to compare the intensity ratio of each chemical function at the two emission angles. The results, presented in Fig. 3e, indicate that the top surface, probably the first layer, is more pyrrolic-like than the “bulk” of the graphene films, which remain more pyridinic-like.

### Architecture of the N-doped graphene films, shown by RAMAN, ARXPS and AES mapping

**Figure 4:**
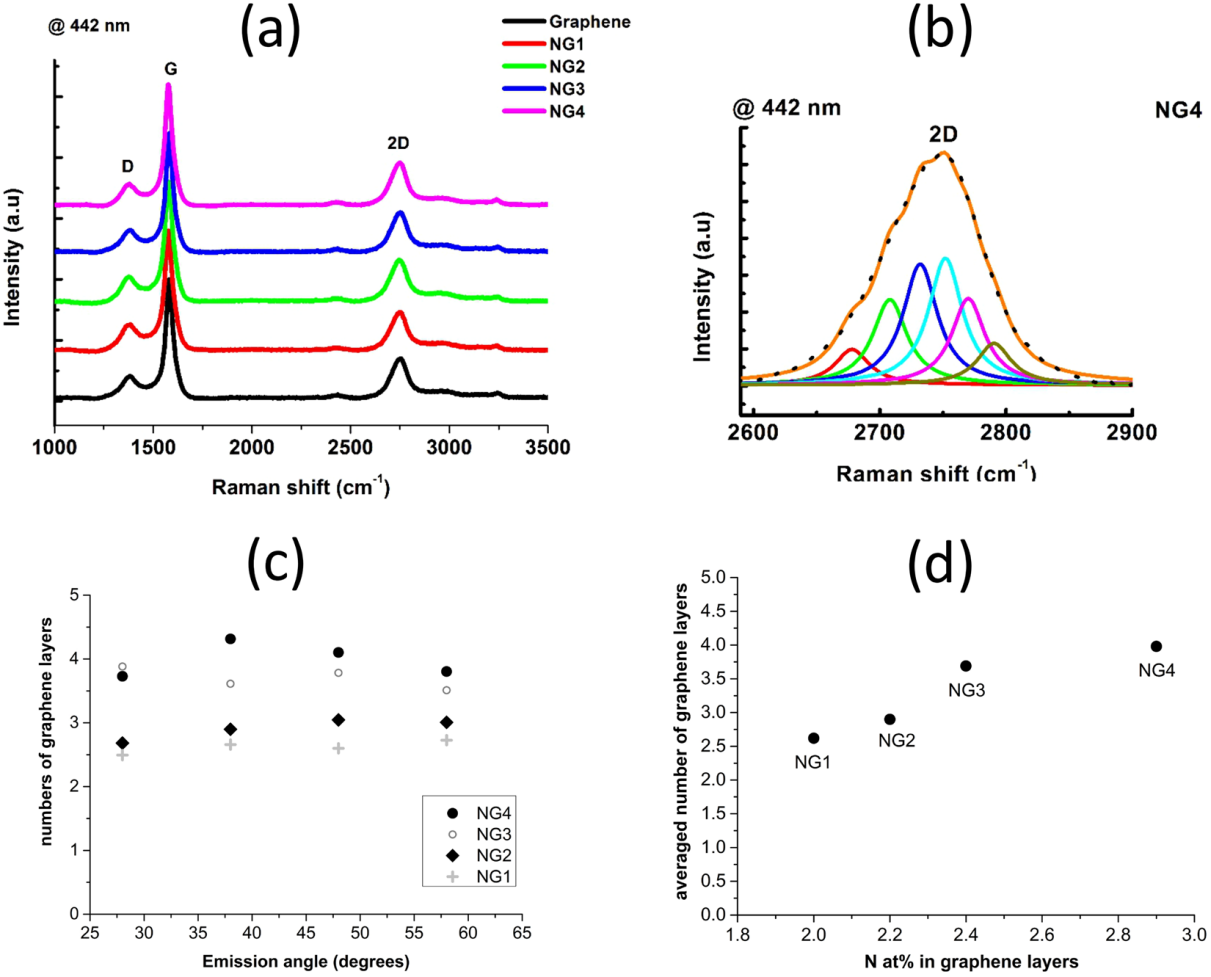
(**a**) RAMAN spectra @ 442 nm of the Graphene and N-doped Graphene films (NG1, NG2, NG3 and NG4 containing respectively 2.0, 2.2, 2.4 and 2.9%N); (**b**) 2D band with deconvolution in six Lorentzian contributions of the NG4 sample (2.9%N); (**c**) number of graphene layers versus the emission angle of photoelectrons; (**d**) average number of graphene layers versus the nitrogen concentration in the N-doped graphene films.

As a primary investigation tool for graphene film architecture, RAMAN spectra, obtained at an excitation wavelength of 442 nm (2.81 eV), are shown in Fig. 4a, which also includes the pure graphene reference.

All spectra displayed three main G, D and 2D bands in rather similar positions with similar shapes, within the 1331–1341 cm^−1^, 1580–1582 cm^−1^ and 2650–2669 cm^−1^ ranges, respectively. Such narrow ranges for each RAMAN band are consistent with strong similarities between the graphene films, and the low nitrogen content of the four N-doped graphene films did not have a significant impact on the general shape of the Raman spectra The G band originates from the first order Raman scattering process and corresponds to the doubly degenerate E_2g_ phonons at the Brillouin zone center. The rather low intense D band originates from the breathing mode of graphitic clusters, and corresponds primarily to the presence of defects in the graphene. This band, characteristic of defects (in-plane substitution nitrogen heteroatoms, vacancies, grain boundaries or edges), is located at half the frequency of the 2D band. Additional RAMAN investigations were conducted at an excitation wavelength of 633 nm (1.96 eV). Whatever the film, the G band remained in the same position compared to 442 nm, whereas both the D and 2D bands underwent a significant blueshift. These average blueshifts, at 633 nm compared to 442 nm, of the D and 2D bands from all synthesis conditions, were respectively +38 and +82 cm^−1^. The dependence of the frequency of both D and 2D bands on excitation energy is consistent with dispersive behavior due to double resonance. According to Malard *et al*.^58^, the D and 2D band frequencies upshift linearly with increasing excitation energy, which is about 50 cm^−1^/eV for the D band and about 100 cm^−1^/eV for the 2D band. Considering the difference of 0.85 eV between our two excitation energies (2.81 and 1.96 eV), such an upshift in energy dependence leads to a blueshift of +42 cm^−1^ for the D band, of +85 cm^−1^ for the 2D band. This is in agreement with our experimental observations of blueshifts of +38 cm^−1^ and +82 cm^−1^, related to our D and 2D bands, respectively. Compared to undoped graphene, nitrogen incorporation led to higher frequencies (blueshift) of the G band (+3–7 cm^−1^), D band (+10–12 cm^−1^) and 2D band (+8–12 cm^−1^). In agreement with Zafar *et al*.^59^, we observed both G and 2D blueshifts, the latter being interpreted as a possible effect of both electron n doping and compressive strain in the graphene sheets, probably due to different thermal expansion of Ni and graphene, as observed by other authors who used copper for graphene synthesis^60^. Additionally, the I(D)/I(G) ratio was not significantly affected by the nitrogen doping: the ratio was 0.298 in the undoped graphene film, and increased slightly from 0.310 to 0.339 with the increase in N content. These ratios are rather low compared to nitrogen doped graphene obtained by other methods^12,61^, but are consistent with crystallite sizes in the 27–30 nm range, according to the Tuinstra-Koenig relation,

$${\rm{La}}({\rm{nm}})=(2.4\cdot {10}^{-10}){\lambda }^{4}{(I(D)/I(G))}^{-1}$$

where λ is the Raman wavelength. The rather stable I(D)/I(G) ratio versus nitrogen doping showed that the nitrogen doping range we investigated did not significantly modify the bonding disorder and vacancies in the graphene sheets whose size remained in the same order of magnitude as that of pure graphene. Thus, the crystallite size of the nitrogen-doped graphene remains rather stable compared to pure graphene. This appears to contradict some previous published works on nitrogen doped graphene, except that is should be recalled that in such studies, the pure graphene used as reference is much more ordered than our films^21,62^. The significant 2D band corresponds to the second order of zone-boundary phonon. The 2D band is sensitive to lattice defects and doping in graphene^63^. In bulk graphite, the 2D band consists of two contributions (2D_1_, 2D_2_) whose height is roughly ¼ and ½ the height of the G peak, forming a dissymmetric 2D band. In pure single sheets of graphene, the 2D peak exhibits only one sharp contribution and can be up to four times more intense than the G band. In the films investigated here, the I(2D)/I(G) intensity ratio started from 0.303 and pure graphene, with a progressive decrease from 0.265 to 0.208 with an increase in N content. This range of I(2D)/I(G) ratios is consistent with the formation of few-layer graphene^41,53,54^, as detailed in the following section. Since the intensity of 2D is depends to a great extent on the electron/hole scattering rate, which is affected by lattice defects as well as by charge carrier doping, our lower I(2D)/I(G) ratio together with higher I(D)/I(G) ratio is consistent with an effect of the charge carrier doping by nitrogen, with no significant effect on the number of lattice defects or disorders. However, the observed decrease in the I(2D)/I(G) ratio and the increase in the I(D)/I(G) ratio are much less than those observed with N-doped graphene obtained by segregation, for a similar range of nitrogen content^21^. The shape of the 2D band of our pure graphene and N-doped graphene films was rather symmetric, which is consistent with a few-layer graphene architecture with weak interlayer coupling. Deconvolution of the 2D bands is known to be a powerful way to quantify the number of graphene sheets, from one monolayer up to about five layers^64^ Such a deconvolution is depicted in Fig. 4b for the N-doped graphene NG4. No significant difference between the nitrogen doped graphene films with different nitrogen contents was observed. The 2D band was affected by the band structures of the material since it arose from the double-resonance process involving transitions among various electronic states. As trilayer graphene has three valence and three conduction bands, up to 15 electronic transitions can contribute to the 2D band^65^. However, many of these different processes have very close energy separations, and it was experimentally shown that the number of Lorentzian functions necessary to correctly fit 2D mode of trilayer graphene is six, each with a FWHM in the 30 cm^−1^ range^64,66,67^. In the studies cited, the graphene investigated was obtained by mechanical cleavage. However, the deconvolution of the 2D bands is also performed in a similar way for graphene films deposited by other methods, including CVD process. For example, Fang *et al*.^68^ also observed a 2D band with a FWHM of 28 cm^−1^ for a single monolayer of graphene, of about 64 cm^−1^ for a bilayer AB stacking, with a decrease in the 2D/G ratio in the latter case. Wei *et al*.^17^ also observed a similar dependence between the 2D band FWHM and the number of layers, with nitrogen doped graphene obtained by CVD. In the present work, the 2D band of all investigated graphene films was successfully deconvoluted into six contributions, each of which with a FWHM of 33 cm^−1^. Thus, we conclude that RAMAN investigations are consistent with a pure graphene and N-doped graphene films constituted of a three-layer architecture, which was confirmed by the AES mapping depicted below. The trilayer graphene films show a rather symmetric 2D peak, which is consistent with Bernal (ABA) stacking, rather than rhombohedral (ABC) stacking, in agreement with the observations reported in^58,59,69^.

**Figure 5:**
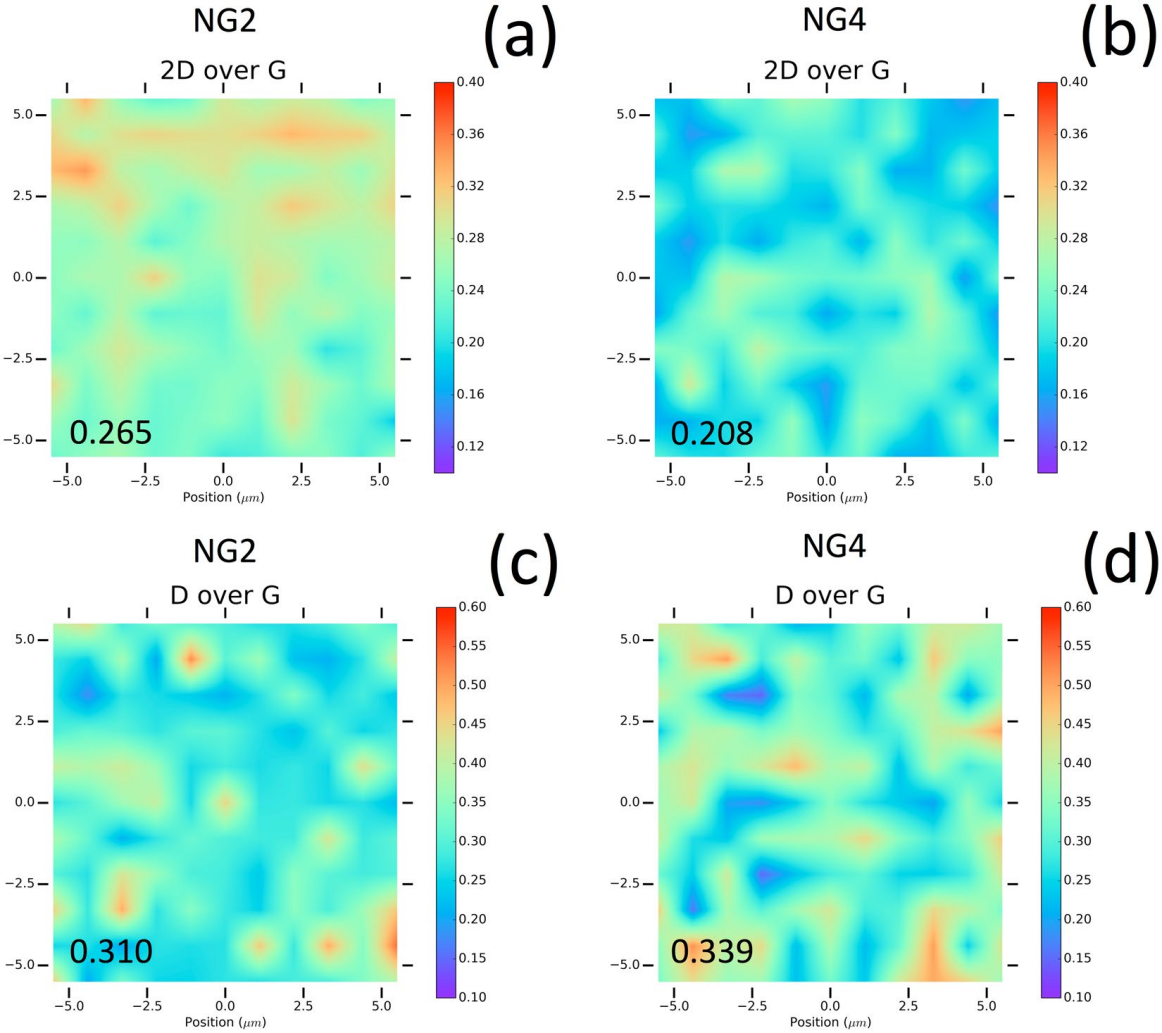
Raman @ 442 nm 2D/G ratio mapping of (**a**) NG2 N-doped graphene (2.0%N) and (**b**) NG4 N-doped graphene (2.9%N); Raman @ 442 nm D/G ratio mapping of (**c**) NG2 N-doped graphene (2.0%N) and (**d**) NG4 N-doped graphene (2.9%N).

Another way to probe film architecture is based on the approach proposed by Tyagi *et al*.^70^ from angular resolved XPS (ARXPS). These authors showed that ARXPS can be used to accurately determine the average thickness of the graphene films, as long as the growth proceeds layer-by-layer. Obviously, their CVD process is not exactly the same as our PLD process, in so far as the carbon species come from the surrounding atmosphere in the former process, and from the Ni catalyst layer in the latter. However, that may be, the methodology, summarized in the Supporting Information, enables determination of the thickness of the graphene, and hence estimation of the number of graphene layers. This approach was compared with the previous RAMAN conclusions obtained by fitting the 2D band. From the ARXPS intensity of a graphene monolayer sample with respect to a highly-ordered pyrolytic graphite (HOPG) sample (which can be approximated as an infinite number of graphene layers), Tyagi *et al*.^70^ quantified the attenuation length of the C1 photoelectrons, depending on the photoelectron emission angle. A stepwise model for the C1 photoelectron intensity was then developed by the authors, which uses the experimentally derived attenuation length, to determine the graphene thickness of a film of arbitrary thickness. Assuming the theoretical thickness of one graphene monolayer (0.335 nm), the method applied to our ARXPS measurements enabled us to deduce the number of graphene layers for each N-doped graphene film with a few percent deviation versus the emission angle, as depicted in Fig. 4c. It is worth noting that calculations were not made for grazing angles >60° in order to minimize roughness and elastic scattering effects. The number of graphene layers averaged over the different electron emission angles were then plotted versus the N concentration in the films, as shown in Fig. 4d. We observed a slight increase in the number of graphene layers, from 2.8 to 4 on average (i.e. at the scale probed by the X-ray probe), with an increase in N content from 2.0 to 2.9 at.%. This quantification of the graphene layer is consistent with the previous RAMAN deduction, and is evidence for slight nitrogen enrichment when a fourth layer is detected, compared to the three-layer architecture. Applications of graphene require control of both lateral and depth heterogeneity depending on the growth mechanism of the films. Even though RAMAN mapping is one of the most popular ways to investigate this characteristic by mapping the D, G, 2D bands and 2D/G, D/G ratios, it does not provide a direct quantification of the graphene depth distribution on the mapped area. The present paper provides a unique and previously unreported possibility to quantify the distribution of the number of monolayers over a scanned surface of the films by AES mapping. Figure 5 depicts both RAMAN and AES mapping related to NG2 (2.2%N) and NG4 (2.9%N) films.

**Figure 6:**
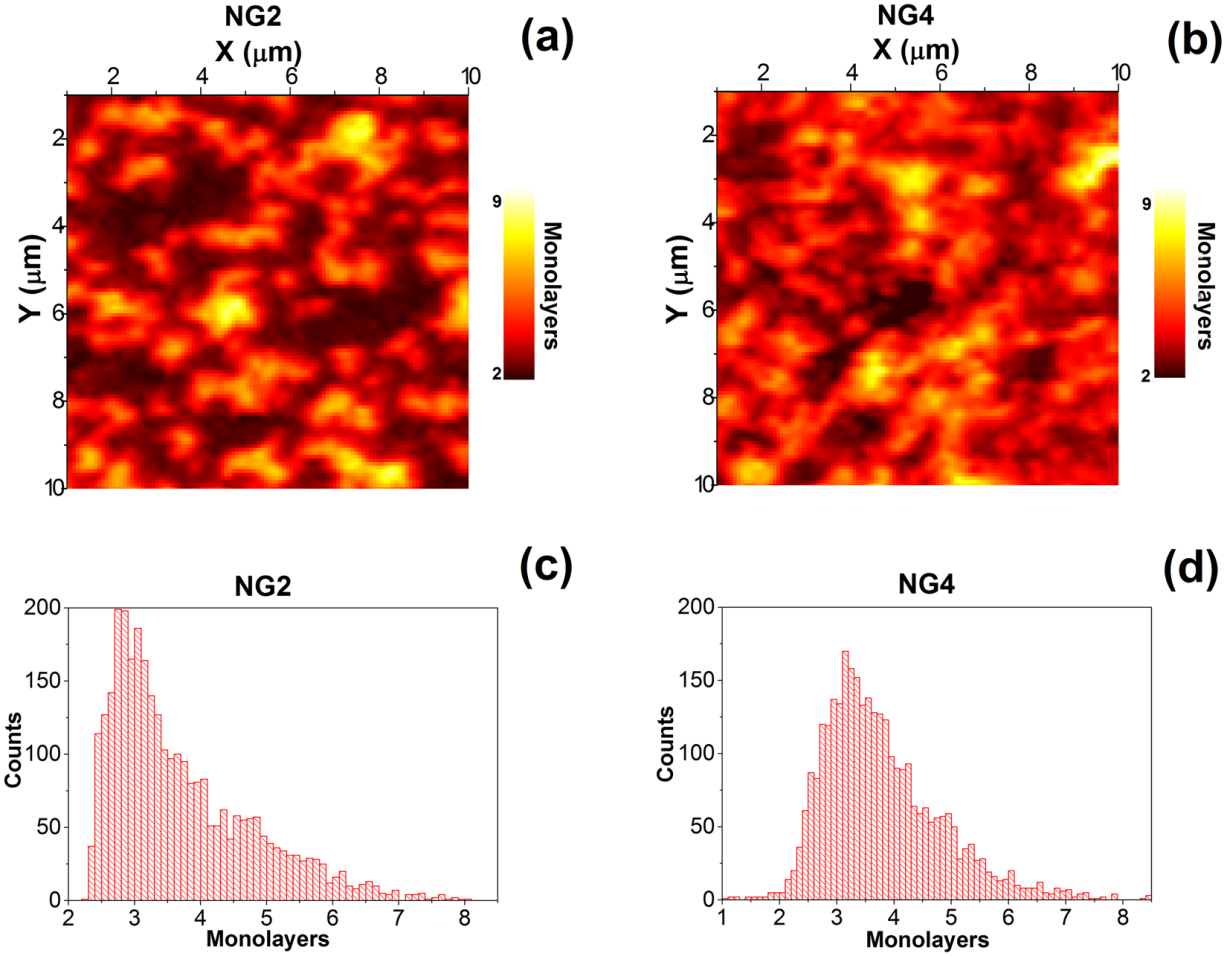
AES mapping of the number of monolayers in (**a**) NG2 N-doped graphene (2.0%N) and (**b**) NG4 N-doped graphene (2.9%N); Distribution of the number of monolayers deduced from AES mapping in (**c**) NG2 N-doped graphene (2.0%N) and ()d NG4 N-doped graphene (2.9%N). Note that both Raman mapping in Fig. 5 and AES mapping were performed on a comparable surface area (10 × 10 μm^2^) but not exactly on the same areas of each sample.

The Raman 2D/G mapping and D/G mapping (@442 nm) of both samples are depicted in Fig. 5a-b-c-d. The bias deposition conditions leading to the highest N content (2.9%) result in a lower 2D/G and a higher D/G, compared to the un-biased condition, which leads to a slightly lower N content (2.2%). This is consistent with the effect of nitrogen substantially deteriorating the intrinsic quality of the graphene (lower 2D/G) with an increase in the doping concentration of nitrogen (higher D/G). Although Raman mapping is traditionally used to qualify the heterogeneity of graphene quality (2D band) and the number of defects (D), the information it provides is limited by the laser beam size, and related to the 2D dimension without any depth quantification within the graphene layers. To investigate in more detail, we optimized an original analytical AES procedure to provide Auger electron mapping of the N-doped graphene surfaces, as depicted in Fig. 6a,b for NG2 and NG4 films.

The 10 × 10 μm^2^ surface was scanned to record 56 × 56 AES spectra per sample, from which the intensity IC_KLL_ (from graphene) and INi_LMM_ (from the catalyst below the graphene layers) were extracted. By using bulk Ni and HOPG references, the intensity ratio of IC_KLL_ to INi_LMM_ was correlated with the number of graphene layers. The resulting AES maps shown in Fig. 6a,b highlight a heterogeneous lateral distribution of N-doped graphene sheets constituted by superimposed graphene layers, with a lateral size in the micrometer scale consistent with the feature revealed by SEM (Fig. 1) and with higher lateral resolution than that obtained with RAMAN. Image analysis of the AES maps enabled us to deduce the statistic of the number of monolayers, as shown in Fig. 6c,d. In both cases, the number of graphene monolayers ranged from 2 to about 6. The distribution of the graphene monolayer number is centered on 3 monolayers, in agreement with RAMAN investigations of the 2D band widths. Since RAMAN mapping areas depicted in Fig. 5 do not correspond exactly to the same areas probed by AES mapping in Fig. 6a,b, such an agreement between RAMAN and AES is a strong indication that areas probed by both analyses are representative of the graphene film architecture. Such a distribution is wider, and slightly shifted toward thicker layers, in the case of the highest nitrogenated (2.9%) NG4 film, compared to the NG2 (2.2%) film, in agreement with the quantification of the number of monolayers obtained with ARXPS depicted in Fig. 4c. However, the correlation between layer distributions and nitrogen contents requires further investigation, since we cannot explain why a significantly higher nitrogen content is associated with only a slight increase in the number of monolayers. Such micrometer-size graphene island observations, with the distribution of monolayer number, appear to be more consistent with an island-on-layer (Stranski-Krastanov) growth mechanism, than a layer-by-layer (Frank-van der Merwe) growth mechanism. The former is certainly more consistent with graphene growth from the underground carbon-based source used in the present investigation, than the later one, which is more consistent with graphene growth from an external carbon source, like with CVD methods. The present set of AES maps offers a powerful new tool to go further in the investigation of the lateral distribution of the number of graphene monolayers, with the high lateral resolution provided by the AES electron probe.

## Conclusion

Femtosecond pulsed laser deposition of solid carbon in an atmosphere of gaseous nitrogen, optionally combined with plasma assistance, gives rise to 10 nm thick amorphous carbon nitride a-C:N films with a nitrogen content of ca. 20 at%. Vacuum heat treatment of this solid source containing carbon and nitrogen, in the presence of a superimposed nickel catalyst film, allows diffusion of carbon and nitrogen into the Ni catalyst, which, after cooling, this leads to the synthesis of self-organized N-doped graphene sheets by precipitation and/or segregation of carbon and nitrogen species on the top surface of the nickel catalyst, in agreement with the diffusion and segregation mechanisms already discussed for graphene synthesis by pulsed laser deposition (PLD)^41^. This robust synthesis route provides a constant and reproducible nitrogen content in doped-graphene films, within 2–3 at.%, whatever the concentration of nitrogen in the a-C:N precursor film, certainly due to the reduced diffusion of nitrogen in the nickel catalyst compared to that in carbon. The introduction of such a low concentration of nitrogen charge carrier does not significantly affect the number of lattice defects and disorders in the graphene sheets compared to pure graphene. It is interesting to note that there is a slight increase in the number of graphene layers with an increase in the concentration of nitrogen in similar thermal annealing conditions. If diffusion of carbon and nitrogen occurs within the grain boundaries of nickel, followed by rapid diffusion onto the surface, probably a slight increase in nitrogen is responsible for the positive co-segregation that enhances the kinetics of formation of the graphene layers. These, to the best of our knowledge, never previously observed results will require investigation in further studies related to the synthesis and characterization of doped graphene. Our experimental analytical investigations represent a significant new methodology to explore the 2D distribution of graphene layers. Auger electron spectroscopy mapping of the N-doped graphene films is an original new way to quantify the number of graphene layers in 2D probed areas. AES mapping was shown to be more quantitative and to have a higher lateral resolution than Raman mapping. AES mapping showed that distribution of the number of layers of the N-doped graphene, synthesized by the thermal annealing of an a-C:N carbon thin layer covered by Ni catalyst, was between 1 and 6, centered around the tri-layer Bernal ABA configuration. Combining ARXPS and XAS quantified four chemical configurations of nitrogen. Although quaternary, pyridinic and pyridinic-oxide nitrogen functions are distributed throughout the whole graphene layer system, pyrrolic nitrogen is predominant in the top layer. The exploration of N-doped graphene synthesis from solid carbon source precursors deposited by femtosecond pulse laser ablation, as well as the analytical investigations focused in particular on AES mapping, extends the perspectives of doped graphene films exploration towards varied functionalities. In particular, our previous studies^34,35,41^ showed that the performance of textured few-layer pure graphene films represent a robust SERS platform for chemical detection, without the need to transfer the film. The present NG films were synthesized using the same route as the pure graphene films, with a similar textured few-layer architecture. One of the next steps in our research will be to advance in SERS probing by incorporating our NG films.

## Methods

### Deposition of a-C and a-C:N films

Ultrasonically cleaned (in acetone then in ethanol baths) SiO_2_ substrates were introduced in a vacuum chamber pumped at a base pressure of 10^−4^ Pa. Amorphous carbon films were deposited by femtosecond pulsed laser deposition at room temperature. A femtosecond oscillator at the 800 nm wavelength, with a pulse duration of 60 fs and a repetition rate of 1 kHz delivered a laser beam focused at an angle of 45° onto a high purity graphite target (99.9995% purity). The energy density (fluence) of the laser beam was kept constant at 5 J/cm^2^. The ablation time was adjusted to keep an a-C and a-C:N film thickness of 10 nm. The SiO_2_ substrates were mounted on a sample holder placed at a distance of 36 mm from the graphite target. During laser ablation, nitrogen gas (99.9995% purity) was used as reactant gas to deposit a-C:N films, and the N_2_ pressure was carefully controlled during deposition using a mass flow controller. Nitrogen pressures were kept constant at 0.5 Pa, 1 Pa and 10 Pa, as listed in Table S1. A nitrogen pressure of 5 Pa was used in combination with an additional DC source to generate a nitrogen plasma interacting with the carbon plasma plume around the growing surface. A negative electrode polarized at −250V was connected to the sample holder. This combination of deposition parameters made it possible to cover a relatively wide range of nitrogen content, 4, 10, 16 and 21 at.% by increasing the pressure of the nitrogen gas, and finally keeping an intermediate nitrogen pressure of 5 Pa with a substrate bias of −250V. Increasing the nitrogen pressure with a similar bias, or modifying the bias values within the experimental range at hand, leads to a significant supplementary increase in the nitrogen content in the film.

### Synthesis of Ni films onto a-C and a-C:N films

Following deposition of the carbon film, a thin nickel film 150 nm thick was deposited by thermal evaporation on the top of the a-C and a-C:N films. High purity (99.99%) Ni was melted thermally in a tungsten nacelle and evaporated towards the substrate. The deposition rate was set at 1.5 nm/minute to minimize residual stress in the growing film, thereby limiting film delamination.

### Vacuum thermal annealing to synthesize graphene and N-doped grapheme

The different a-C/Ni/SiO_2_ and a-C:N/Ni/SiO_2_ films were heated from room temperature to 780 °C at a heating ramp rate of 4 °C/s, then maintained at 780 °C for 30 minutes in a vacuum pressure of 10^−4^ Pa. The vacuum pressure was maintained during natural cooling to room temperature. The vacuum was broken a minimum of 5 hours after the end of the heating stage.

### XPS/AES analysis

X-Ray photoelectron spectroscopy (XPS) and Auger electron spectroscopy (AES) were performed with a Thermo VG Thetaprobe spectrometer (Thermo Fisher Scientific). XPS analysis was carried out with a focused monochromatic AlKα source (hν = 1486.68 eV, 400 µm spot size) while a field emission electron gun with 150 nm spotsize operating at 10 kV accelerating voltage and 5 nA beam current was used for AES analysis. Electrons were analyzed using a concentric hemispherical analyzer operating in the constant ∆E mode for XPS analysis and fixed retard ratio mode for AES analysis. The energy scale was calibrated with sputter-cleaned pure reference samples of Au, Ag and Cu such that Au4f_7/2_, Ag3d_5/2_ and Cu3p_3/2_ were positioned at binding energies of respectively 83.98, 386.26 and 932.67 eV. In the case of XPS analysis, for all the samples analyzed, narrow scans were recorded for C1s, O1s and N1s with a step size of 0.1 eV and pass energy of 50 eV. This pass energy gives a width of the Ag3d_5/2_ peak measured on a sputter clean pure Ag sample of 0.55 eV. Components in C1s and N1s peaks were adjusted using line shapes consisting of a convolution product of a Gaussian function (75%) and Lorentzian function (25%), except for Csp^2^ hybridized and Csp^3^ hybridized components of C1s peak for which asymmetric line shapes were used and adjusted on HOPG and graphene synthetized samples. The C1 and N1s components related to the various films are listed in Table S2 and Table S3. The nitrogen concentrations in the amorphous carbon film source and in the synthetized nitrogen doped graphene film were quantified using equation 1 below using sensitivity factors F, considering the calibrated transmission function of the spectrometer, the Scofield ionization cross section of C1s and N1s core levels and the electron attenuation length of electrons coming from the C1s and N1s:

1 $${C}_{A}( \% at)=\frac{{I}_{A}/{F}_{A}}{{\sum }_{n}{I}_{n}/{F}_{n}}$$

The Auger mapping procedure is detailed in Supporting Information.

### Angular Resolved XPS

The average thickness of graphene films was determined with angle-resolved X-ray spectroscopy measurements using the formalism of Tyagi *et al*.^70^. It is worth noting that our measurements were acquired using a type of spectrometer that simultaneously collects several photo-electron emission angles over a 60° range without tilting the sample. The step-wise growth model proposed by Tyagi *et al*. is based on two assumptions: (i) the graphene film covers the entire surface, (ii) the growth mode is assumed to be layer-by-layer. The procedure is detailed in Supporting Information.

### High resolution soft X-ray absorption near-edge spectroscopy

XANES spectra were recorded at the ANTARES beam line specially designed to perform complementary photoemission and X-ray soft absorption, at the SOLEIL Synchrotron (Saclay, France). The ring operating conditions were 2.5 GeV electron energy, with injection currents of 500 mA and “Top-up” mode. Radiation was monochromatized using a plane-grating monochromator (PGM), which is characterized by a slitless entrance and the use of two varied linear spacing (VLS) gratings with variable groove depth (VGD) along the grating lines. All measurements were performed at 20.0(2) °C for C K-edge and N K-edge over the range 280–315 eV and 380–420 eV, respectively, with a step size of 0.2 eV to enable correct normalization of the XANES spectra. The absorption data were acquired in the partial electron yield (PEY) mode by collecting X-ray electron emissions from the sample with a Scienta R4000 analyser. The XAS spectra of the samples were normalized to the signal from a gold covered grid recorded simultaneously. The resolution of the beam line at the C and N K-edge was 50 meV.

### RAMAN spectroscopy

Raman spectroscopy was performed using an Aramis Jobin Yvon spectrometer at both 633 nm (1.96 eV) and 442 nm (2.81 eV), with a spectral resolution of 2 cm^−1^. The laser beam was focused on the sample with a 100x objective and the laser power was kept below 3 mW whatever the excitation wavelength, to avoid damaging the surface of the film. The diameter of the laser beam was estimated to be near 1 micrometer, near the diffraction limit at this wavelength. RAMAN maps were built from the acquisition of each 1 micrometer spectrum, over probe surfaces 10 × 10 micrometers in size. The Raman signals were acquired by a spectrometer equipped with a charge coupled device (CCD) camera. Raman spectra were deconvoluted by symmetric Lorentzian functions. Only the peak positions and intensities were kept free by the fitting process.

## Electronic supplementary material

Supplementary Information
