# Supplementary material for: Nano-Architecture of nitrogen-doped graphene films synthesized from a solid CN source
Source: Sci Rep. 2018 Feb 19;8:3247. doi: 10.1038/s41598-018-21639-9 (PMC5818613; doi:10.1038/s41598-018-21639-9)
Supplement: Supplementary file 1 — Supplementary Information [file 41598_2018_21639_MOESM1_ESM.pdf]

Supplementary Information

Nano-Architecture of nitrogen-doped graphene films synthetized from a solid CN source

Chiranjeevi MADDI(1), Florent BOURQUARD(1), Vincent BARNIER(2), José AVILA(3), Maria-Carmen ASENSIO(3), Teddy. TITE(1), Christophe DONNET(1), Florence GARRELIE(1)

(1) Univ. Lyon, Laboratoire Hubert Curien UMR 5516 CNRS, Université Jean Monnet, F-42000 Saint-Étienne, France

(2) Laboratoire Georges Friedel, Ecole Nationale Supérieure des Mines, F-42023 Saint-Etienne, France

(3) Synchrotron SOLEIL & Université Paris-Saclay, Saint Aubin, F-91192 Gif sur Yvette, France

Corresponding author: Pr Christophe DONNET

Email: Christophe.Donnet@univ-st-etienne.fr

Table S1 Synthesis conditions and nitrogen contents

Label	Sample	N2 pressure (Pa)	DC bias (V)	N at.% a-C:N	N at.% N-doped graphene
**Graphene**	a-C / Ni / SiO_2	0	0	0	0
**NG1**	a-C:N / Ni / SiO_2	0.5	0	4	2.0
**NG2**	a-C:N / Ni / SiO_2	1	0	10	2.2
**NG3**	a-C:N / Ni / SiO_2	10	0	16	2.4
**NG4**	a-C:N / Ni / SiO_2	5	-250 V	21	2.9

Table S1: The depositions conditions and N concentrations deduced from XPS in the a-C:N films (before Ni thin film deposition and thermal annealing) and in the N-doped graphene. The thicknesses of the a-C(:N) and Ni films are systematically 10 nm and 150 nm, respectively. One sample without N incorporation is used as reference.

Table S2 Chemistry of carbon in the graphene films

C1s component (%)	C_{Gr}	C_B	C_{Dis}	C_N	C_O
**Graphene**	69	29	2	-	-
**NG1**	41	39	6	11	3
**NG2**	41	42	5	10	3
**NG3**	44	40	4	10	2
**NG4**	45	37	4	11	3

Table S2: The assignation of the C1s components from XPS are based on [1].

Table S3 Chemistry of nitrogen in the graphene films

N1s component (%)	N pyridinic	N pyrrolic	N quaternary	N pyridinic oxide
**NG1**	35	17	20	28
**NG2**	41	14	24	21
**NG3**	36	17	22	25
**NG4**	38	16	21	25

Table S3: The assignation of the N1s components from XPS are based on [1].

Angular-Resolved XPS applied to the quantification of the graphene layers number

Considering these two assumptions and according to work of Tyagi et al.[2], the total intensity of the C1s peak can be written as follow:

$$I_T = I_{SLG} + I_{2L} + ... + I_F \qquad (2)$$

where I_{SLG} is the intensity from a single layer graphene, I_{nL} is the intensity from the n layer graphene and I_F is the intensity from the fractional layer on the top of the graphene film.

The intensities I_{SLG} and I_{nL} can be described by the following equations 3 and 4:

$$I_{SLG} = I_{HOPG} \times \left[1 - \exp\left(\frac{-d_{SLG}}{\lambda_{C1s}^{graphene} \cos\theta} \right) \right] \qquad (3)$$

$$I_{nL} = I_{HOPG} \times \left[e^{-\frac{(n-1)d_{SLG}}{\lambda_{C1s}^{graphene} \cos\theta}} - e^{-\frac{n d_{SLG}}{\lambda_{C1s}^{graphene} \cos\theta}} \right] \qquad (4)$$

where $d_{SLG} = 3.35$ Å is the thickness of one monolayer, I_{HOPG} the intensity of C1s measured on a pure HOPG standard sample and $\lambda_{C1s}^{graphene}$ the electron attenuation length in graphene. For $\lambda_{C1s}^{graphene}$, we chose the values dependent on electron take-off angles measured by Tyagi et al. on single layer graphene grown on Cu foil. For the four electron take-off angles in the range of 28° to 58° used to estimate the average thickness of graphene films, the electron attenuation length varies slightly from 1 nm to 1.4 nm.

Then, following the step-wise growth model, the average thickness of graphene films is calculated for each electron take-off angle iteratively using the steps indicated below:

if $I_T - I_{SLG} < I_{2L}$ then number of layers $= 1 + \frac{I_T - I_{SLG}}{I_{2L}}$ with $I_F = \frac{I_T - I_{SLG}}{I_{2L}}$

if $I_T - I_{SLG} - I_{2L} < I_{3L}$ then number of layers $= 2 + \frac{I_T - I_{SLG} - I_{2L}}{I_{3L}}$ with $I_F = \frac{I_T - I_{SLG} - I_{2L}}{I_{3L}}$

....

To check the first assumption, the narrow scans of $Ni2p_{3/2}$ peak at different photoelectron emission angles were carried out. The **Figure S1** shows $Ni2p_{3/2}$ spectra acquired on the sample NG4 at an emission angle close to the normal of the sample figure 1a) and for a grazing angle figure 1b). In the case of grazing angle, the component with a shape derived from measurement on pure ion cleaned Ni standard sample does not describe correctly the experimental shape of $Ni2p_{3/2}$ spectrum. Two other components consisting in principal peak with an associated satellite were added in order to fit the shape of the $Ni2p_{3/2}$ spectrum. The adjustment to the experimental spectrum gave a ratio of the intensities of satellite to principal peak of 0.4 as well as a shift of 5.6 eV between these peaks which corresponds to $Ni(OH)_2$ as referred to [3]. If we assume that $Ni(OH)_2$ is formed at room temperature where Ni is not covered by graphene layers, the surface covering ratio of $Ni(OH)_2$ is good indicator of the graphene film coverage.

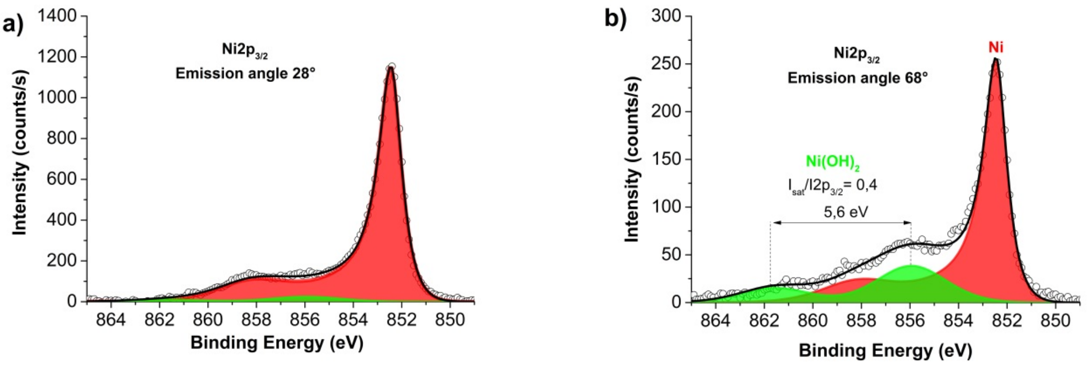

Figure S1: $Ni2p_{3/2}$ peak measured on NG4 sample for two photoelectron emission angles with respect to the normal of the sample: a) 28° and b) 68°.

The two components associated to $Ni(OH)_2$ determined at the electron emission angle of 68° were used to adjust the $Ni2p_{3/2}$ spectra at others photoelectron emission angles. The intensities ratio of Ni and $Ni(OH)_2$ components can be then described as:

$$\frac{INi2p_{3/2}^{Ni(OH)_2}}{INi2p_{3/2}^{Ni}}=\frac{\frac{\lambda_{Ni2p_{3/2}}^{Ni(OH)_2}}{\lambda_{Ni2p_{3/2}}^{Ni}}\times\alpha\times\left(1-\exp\left(\frac{-d^{Ni(OH)_2}}{\lambda_{Ni2p_{3/2}}^{Ni(OH)_2}\cos\theta}\right)\right)}{(1-\alpha)\times\exp\left(\frac{-d^{graphene}}{\lambda_{Ni2p_{3/2}}^{graphene}\cos\theta}\right)+\alpha\times\exp\left(\frac{-d^{Ni(OH)_2}}{\lambda_{Ni2p_{3/2}}^{Ni(OH)_2}\cos\theta}\right)} \quad (5)$$

where α is the surface coverage of $Ni(OH)_2$, $d^{Ni(OH)_2}$ the thickness of $Ni(OH)_2$ film, $d^{graphene}$ the thickness of the graphene film determined with the stepwise model, $\lambda_{Ni2p_{3/2}}^{Ni(OH)_2}$, $\lambda_{Ni2p_{3/2}}^{Ni}$ and $\lambda_{Ni2p_{3/2}}^{graphene}$ the attenuation lengths of electron coming from the $Ni2p_{3/2}$ core level and travelling respectively through $Ni(OH)_2$, Ni and graphene materials. $\lambda_{Ni2p_{3/2}}^{Ni(OH)_2}$ and $\lambda_{Ni2p_{3/2}}^{Ni}$ were determined with the TPP2M method [4] and gave respectively 1.3 and 1.1 nm and $\lambda_{Ni2p_{3/2}}^{graphene}$ was estimated to 1.1 nm [5]. **Figure S2** presents the result of the adjustment of the theoretical ratio given by equation 4 and the experimental which are in good accordance. A 3% surface coverage of $Ni(OH)_2$ was found which suggests that the graphene film covers almost entirely the surface and validates the first assumption. Similar results were found for the others nitrogen doped graphene films.

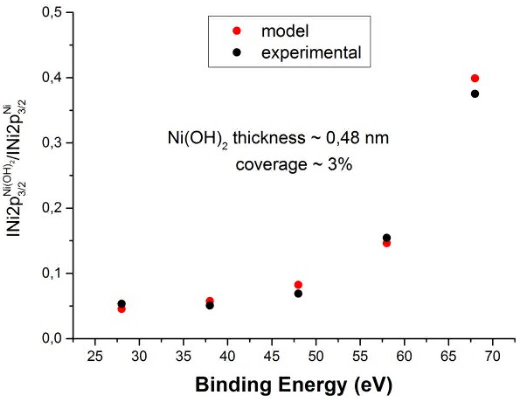

Figure S2: experimental and theoretical intensities ratio of Ni2p$_{3/2}$ peak for Ni and Ni(OH)$_2$ in function of the photoelectron emission angle. The adjustment of the model given by equation 4 gave a Ni(OH)$_2$ film with a thickness of 0.48 nm and a surface coverage of 3%.

Auger Electron Spectroscopy Mapping

AES mapping of nitrogen doped graphene films were performed on 10x10 μm^2 area with scanning rate of 56x56 points. For each point, narrow scans of C KLL and Ni $L_3M_{4,5}M_{4,5}$ transitions were sequentially recorded. These transitions were also acquired on HOPG and pure Ni standard samples in equivalent experimental conditions. As shown in the **Figure S3**, the spectra were after acquisition numerically derived and the intensities of each transitions were measured as a peak to peak eight.

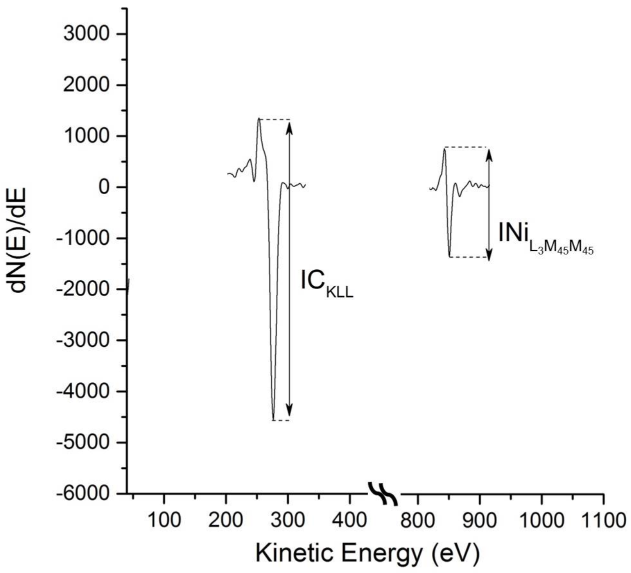

Figure S3: measurement of the intensities of C KLL and Ni $L_3M_{4,5}M_{4,5}$ transitions after derivation of experimental narrows scans. Each narrow scan were sequentially recorded for each point of the Auger Mapping on an area of 10 X 10 μm^2 with a scan rate of 56 X 56 points.

Considering a model of a substrate with an overlayer film, the ratio of the intensities of C KLL and Ni $L_3M_{4,5}M_{4,5}$ can be expressed as:

$$\frac{I_{C_{KLL}}}{I_{Ni_{L3M45M45}}} = \frac{I^{std}{}_{C_{KLL}}}{I^{std}{}_{Ni_{L3M45M45}}} \times \frac{1-\exp\left(-\frac{n\times d^{SLG}}{\lambda_{C_{KLL}}^{graphene}}\right)}{\exp\left(-\frac{n\times d^{SLG}}{\lambda_{Ni_{L3M45M45}}^{graphene}}\right)} \qquad (6)$$

where $d_{SLG} = 3.35\ \overset{\circ}{A}$ is the thickness of one graphene monolayer, $I^{std}{}_{C_{KLL}}$ and $I^{std}{}_{Ni_{L3M45M45}}$ the intensities measured on HOPG and pure Ni standard samples and n the numbers of graphene layers. The attenuation lengths of electron coming from C KLL and Ni $L_3M_{4,5}M_{4,5}$ transitions and travelling through graphene layers were taken as $\lambda_{C_{KLL}}^{graphene} = 7$ Å and $\lambda_{Ni_{L3M45M45}}^{graphene}$ = 14Å according to [4]. For each points of the Auger mapping, the number of graphene layers was then

calculated iteratively until the theoretical intensities ratio given by equation 6 matches the experimental one.

---

1. R.S. Weathertrup, B.C. Bayer, R. Blume, C. Ducati, C. Baehtz, R. Schlögl, S. Hofmann, In situ characterization of alloy catalysts for low-temperature graphene growth, NanoLetters 11 (2011) 4154-4160.

2. P. Tyagi, Z.R. Robinson, A. Munson, C.W. Magnuson, S. Chen, J.D. McNeilan, R.L. Moore, R.D. Piner, R.S. Ruoff, C.A. Ventrice Jr, Characterization of graphene films grown on CuNi foil substrates, Surface Science 634 (2015) 16-24.

3. S. Diplas, T. Knutsen, S. Jørgensen, A. E. Gunnæs, T. Våland,T. Norby, A. Olsend and J. Taftød, An experimental study of the electronic structure of anodically grown films on an amorphous Ni78Si8B14 alloy, Surf. Interface Anal. 40 (2008) 826–829.

4. S. Tanuma, C.J. Powell, D.R. Pen, Calculations of electron inelastic mean free paths (IMFPS). IV. Evaluation of calculated IMFPs and of the predictive IMFP formula TPP-2 for electron energies between 50 and 2000 eV, Surface and Interface Analysis, 20 (1993) 77-89.

5. C. Martin, E.T. Arakawa, T.A. Callcott, R.J. Warmack, Attenuation lengths of low-energy electrons in free-standing carbon films, Journal of Electron Spectroscopy and Related Phenomena 42 (1987) 171-175.
